# Natural distinction of carbon and nitrogen isotopic niches in common fish species across marine biotopes in the Yellow River estuary

**DOI:** 10.1002/ece3.7895

**Published:** 2021-07-09

**Authors:** Pei Qu, Min Pang, Fangyuan Qu, Zhao Li, Meng Xiao, Zhaohui Zhang

**Affiliations:** ^1^ Observation and Research Station of Bohai Eco‐Corridor First Institute of Oceanography Ministry of Natural Resources of the People's Republic of China Qingdao City China; ^2^ Pilot National Laboratory for Marine Science and Technology (Qingdao) Qingdao City China; ^3^ China National Environmental Monitoring Centre Beijing City China; ^4^ Qingdao University of Science & Technology Qingdao City China

**Keywords:** estuarine offshore biotope, fish community, food source, stable isotopic niche, trophic level

## Abstract

Stable isotope analysis is a universally recognized and efficient method of indicating trophic relationships that is widely applied in research. However, variation in stable isotope ratios may lead to inaccuracies due to the effects of complex environmental conditions. This research compared the carbon and nitrogen isotopic niches of fish communities between diverse biotopes around the Yellow River estuary and adjacent sea areas, with the aim of revealing distinctions in stable isotopic niche metrics, trophic positions, and feeding preferences. Our analysis of the food source contribution indicated that allochthonous sources were considered major energy sources in estuarine areas directly affected by Yellow River‐diluted water, while autochthonous benthic and pelagic producers dominated carbon input into the food web in Laizhou Bay and the open water. A significant variation in the fish δ^15^N characteristic was found within estuarine adjacent regions, so, together with the results from previous studies, we deemed the local high concentration of dissolved inorganic nitrogen as the original trigger of the abnormal δ^15^N characteristic in fishes via a transport process along food chains. These results provide a new perspective on the natural distinction of carbon and nitrogen isotopic niches. The detailed data reported here enhance our understanding of variations in fish communities in estuarine ecosystems.

## Graphical abstract

Our research aimed to compare the isotopic niches of fish communities between diverse biotopes in the Yellow River estuary with the main expectation of revealing distinctions in stable isotopic niche metrics, trophic positions, and feeding preferences. These results provide new perspectives on the natural distinction of carbon and nitrogen isotopic niches, and they provide detailed data that can enhance our understanding of the variations in fish communities in estuarine ecosystems, with important implications for fishery conservation and the restoration of estuarine marine protected areas.

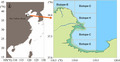

## 1 INTRODUCTION

Estuarine biotopes display distinct trophic structures of biocoenosis driven by the supply and transformation of multiple energy sources (Underwood, 2010), while flowing waters sustain riverine and marine biodiversity, and make important contributions to global biogeochemical cycles (Palmer & Ruhi, 2019). It has been widely recognized that most adjacent marine ecosystems are strongly connected due to the water transference of organic matter and bioelements (Stasko et al., 2018; Sujitha et al., 2019). Adjacent ecosystems also provide ecological corridors for animal migration (Hastie et al., 2016). Numerous investigations have shown that transference of energy and materials occurs frequently between biotopes influenced by strong coastal physical and biological dynamics (Livernois et al., 2019). This implies the potential connectivity of trophic niches and biocoenosis structures (Palmer & Ruhi, 2019). However, there is relatively little research on identifying discrepancies in fish trophic niches caused by diverse marine biotopes around the estuary directly. These related studies are limited to temporal and spatial heterogeneity in food sources (McMahon et al., 2015) and the complexity of marine ecosystems within various biotopes (Christianen et al., 2017; Ramshaw et al., 2017). Kratina et al. (2014) used multivariate autoregressive models with detailed time series data from largely freshwater and brackish regions of the upper San Francisco Estuary to assess trophic interactions. However, our understanding of these interactions is still inadequate, which hinders our understanding of the energy and material transporting mechanisms in food webs.

Stable isotopes record information of marine lives accumulating nutrients over integrated time periods in lifecycles as opposed to a snapshot of food ingestion (Plass‐Johnson et al., 2013). They can be used to reconstruct the trophic structures of biocoenoses in marine food webs (Boecklen et al., 2011; Fry, 2013; Parnell et al., 2010). Primary producers often differ in terms of carbon isotope values (δ^13^C) due to the variety of carbon sources and fractionation during photosynthesis (Christianen et al., 2017). For example, C_3_ and C_4_ plants exhibit a discriminative carbon isotopic characteristic (Warne et al., 2010). Thus, we can identify the primary consumers according to their δ^13^C values. Controlled laboratory experiments that verify nitrogen stable isotope values (δ^15^N) can be converted to direct estimates of trophic levels (TLs) using assumed values of discrimination in trophic transfers in food webs (Caut et al., 2010; Hussey et al., 2014; Layman et al., 2012; Reum et al., 2015). However, mixed models that take isotope values of multiple food sources into account according to user‐specified data have been developed and successfully applied to food web studies to solve complex interpretation processes (Jackson et al., 2011; Phillips, 2012). Stable isotopic analysis can greatly contribute to research on fish community connectivity in marine ecosystems on account of isotopic signatures corresponding to estuarine biotopes (Selleslagh et al., 2015). As the trophic relationship can be concisely expounded using stable isotope analysis together with advisable models, such as SIBER (Stable Isotope Bayesian Ellipses in R, Jackson et al., 2011) and IsoSource (Layman et al., 2012), a comparison of trophic relationships of biocoenoses between diverse biotopes, including variations in trophic structures, can be further indicated.

The Yellow River is the second longest river in China, and its input routes have changed over time, leading to complicated biotopes in the estuary (Xu et al., 2013). There is a strong interaction between ocean and land as with many large river estuaries, and the research value is significant in terms of the diverse marine food webs within the Yellow River estuary. However, to date, few studies have compared the trophic relationships between diverse biotopes in Yellow River estuarine ecosystems. This shortage of information hinders our understanding of the energy and materials transportation mechanism in local food webs and impedes the restoration and conservation process in estuarine Marine Protected Areas (MPAs).

This research aimed to compare the isotopic niches of fish communities between diverse biotopes in the Yellow River estuary with the main expectation of revealing distinctions in stable isotopic niche metrics, trophic positions, and feeding preferences. These results provide new perspectives on trophic relationships, and they provide detailed data that can enhance our understanding of the variations in fish communities in estuarine ecosystems, with important implications for fishery conservation and the restoration of estuarine MPAs.

## 2 METHOD

### 2.1 Research area and sampling methods

**FIGURE 1:**
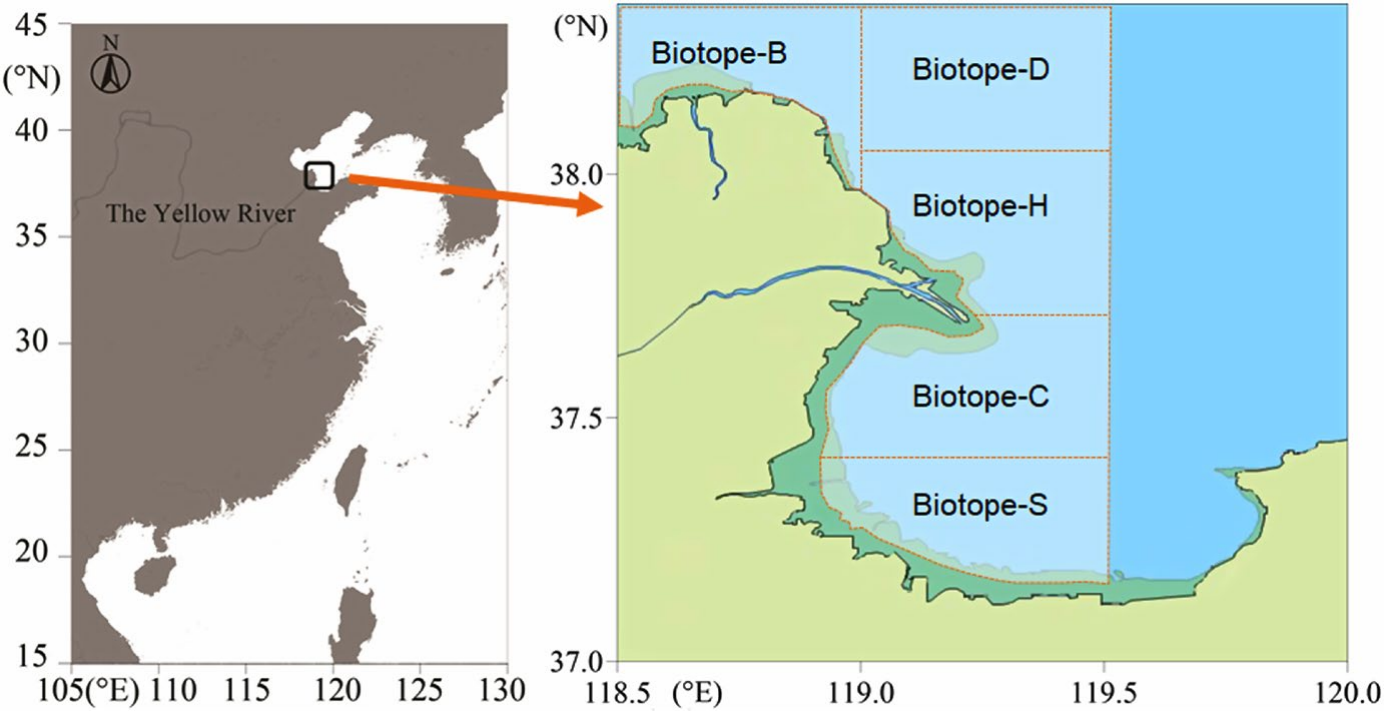
Research area off the Yellow River estuary and adjacent sea areas

Two sampling cruises were launched in September 2017 and September 2018. Study areas were the coastal sea located from 118.5°E to 119.5°E and 37.0°N to 38.4°N around the Yellow River estuary (Figure 1). The location included five MPAs: Yellow River Estuary MPA, Lijin MPA, Hekou MPA, Laizhou MPA, and Guangrao MPA. MPAs are considered ideal experimental systems in which to obtain environmental background values in areas that restrict human activities. The inherent variety of fish communities in a stable isotopic niche caused by a diverse marine biotope can be compared with different biotopes that exclude direct human exploitation.

Selection of survey biotopes mainly considered the diversity between typical estuarine and gulf ecosystems, as well as subtidal zones and deeper water. Five biotopes (Figure 1) with unique individual characteristics were selected. All five biotopes were separated by the Yellow River estuary and differentiated by intertidal and subtidal zones. They represented a salinity gradient around the Yellow River estuary, which was synthetically used to distinguish the influencing scope of diluted water from the river. Each of these five biotopes had discriminative marine ecosystem characteristics. Biotope‐H, located at the intertidal and subtidal zone of the recent Yellow River estuary, was directly affected by the abundant diluted water from the Yellow River and characterized by typical estuarine features, such as abundance of terrestrial organic matters, lower salinity, and depth. Biotope‐C was located at the intertidal and subtidal zone of the southern branch of the Yellow River estuary that was almost blocked in the 1990s. Biotope‐D was located at the subtidal zone facing open water and featured deeper water and higher salinity. Biotope‐B was located at the intertidal and subtidal zone near the ancient Yellow River estuary facing open water. Biotope‐S, which was located at the intertidal and subtidal zone in Laizhou Bay, was closer to the mainland and influenced more by the weaker diluted water from the bottom of Laizhou Bay rather than diluted water from the Yellow River. Five evenly spaced sites were chosen from each biotope for comparison; thus, in total, twenty‐five sites were selected for collecting fish specimens for stable isotopic analysis. After the summer fishing moratorium ended in September 2017 and September 2018, a fishing boat trawled at 2 kn for 30 min (Choy et al., 2015) at each site. Specimens were weighed and frozen at −20℃ after species identification. Three replicates of stable isotope analysis were carried out on each species. Zooplankton specimens were collected as an assemblage of communities using a 200‐µm zooplankton net that was horizontally trawled at 2 kn for 10 min. Three replicate samples were taken, filtered using a pump system and 200‐µm bolting silk filters, wrapped in foil, placed in sealed bags, and then stored at −20℃ until further analysis. Suspended particulate organic matter (POM), which mainly contained phytoplankton and organic detritus, was collected by filtering seawater through precombusted Whatman GF/F glass fiber filters (Kohlbach et al., 2016), and subsequent methods were the same with zooplankton sample collection. Microscopic photosynthetic organisms living on the sediment surface were referred to as microphytobenthos, mainly comprising diatoms and cyanobacteria (Christianen et al., 2017), which compose the sedimental organic matter (SOM) with other organic detritus. SOM specimens were collected using a clam grab bucket. The surface layers (<5 mm) of the sediment were scraped off, foil‐wrapped, and then frozen at −20℃ for further processing. Enteromorpha and spartina were, respectively, collected and then foil‐wrapped and frozen as a proxy for macroalgae and cordgrass.

### 2.2 Sample treatment and stable isotope analysis

In this study, local dominant fish species from the five research biotopes were collected for δ^13^C and δ^15^N analysis. Common fish species were defined as those with high biomass, and their combined biomass accounted for more than 90% of the total biomass of collected fish. The dorsal muscle tissues of fishes were used for stable isotope analysis, which reflects long‐term information about nutrient accumulation (McIntyre & Flecker, 2006). Mixed zooplankton samples composed of several species were analyzed together to gain nitrogen stable isotope data for the TL baseline (Hoen et al., 2014). Each specimen was separated into two equal quantity samples. One sample was treated with 1 mol/L hydrochloric acid to remove inorganic carbon for δ^13^C analysis (Kanaya et al., 2007), while the nonacidified one was used directly for δ^15^N analysis. All samples were oven‐dried for approximately 24 to 48 hr at 70℃ until a constant weight was achieved and then homogenized into uniform particle size powder using a triturator.

After pretreatment, the main process of carbon and nitrogen isotopic analysis was performed using an isotope ratio mass spectrometer (Delta V™, Thermo Fisher). Stable isotope ratios were expressed in standard δ unit notation (δ^13^C and δ^15^N), and defined as follows:

(1) $$\delta^{13}C\left(‱\right)=\left(\frac{{}^{13}C/{}^{12}C_{s}}{{}^{13}C/{}^{12}C_{\text{VPDB}}}‐1\right)\times 1000$$

(2) $$\delta^{15}N\left(‱\right)=\left(\frac{{}^{15}N/{}^{14}N_{s}}{{}^{15}N/{}^{14}N_{\text{air}}}‐1\right)\times 1000$$

where ^13^C/^12^C_s_ and ^15^N/^14^N_s_ are the ratios of heavy isotopes to light isotopes from the samples and ^13^C/^12^C_VPDB_ and ^15^N/^14^N_air_ are the Vienna Pee Dee Belemnite (VPDB) standard and atmospheric N_2_ standard for ^13^C and ^15^N, respectively (Jeglinski et al., 2013).

### 2.3 Data analysis

δ^13^C and δ^15^N were analyzed using the current most efficient procedures, including SIBER metrics (Stable Isotope Bayesian Ellipses in R version 3.6.1), SIAR package (Stable Isotope Analysis in R), and the TL model (Du et al., 2020; Post, 2002). SPSS Statistics Subscription (IBM Inc.) was also used to determine how similar isotopic signatures were and to distinguish the sources of identical stable isotope characteristics. Differences in calculated results were compared with the *t* test and one‐way analysis of variance statistical method.

### 2.4 Isotopic niche analysis

SIBER is a multivariate ellipse‐based model available in an R statistical computing package, which can reformulate metrics in a Bayesian framework for direct comparison of isotopic niches across biocoenosis (Jackson et al., 2011). When comparing individual groups with each other, either within a single community or in groups of communities, the Standard Ellipse Area (SEA) was recommended by the program author. With the SIBER object created, isotope biplots could be displayed using stated functions, and some summary statistics could be calculated for each group in the dataset. In this study, a pairwise comparison of biotopes was implemented using SIBER. The stable isotopic niche areas of each group, which were determined by the SEA, represented the trophic niche of respective fish communities plotted on a δ^13^C‐δ^15^N dot plot. The ellipse area corrected for the small sample size (SEAc) and the stable isotopic niche width of each fish community was computed for comparison.

### 2.5 Trophic level calculation

Seven common fish species were collected to calculate and compare their TLs in each biotope. TLs were determined based on the nitrogen isotopic fractionation for ^15^N enrichment through the food chains considering the consumer ingestion and metabolic process (Caut et al., 2009, 2010), undergoing predictable changes with each successive level up the trophic ladder (Smit et al., 2005). The recognized trophic fractionation factor of *δ* ^15^N (Δ^15^N) was 3.4‰ between contiguous TLs (Post, 2002). TLs could be calculated using the traditional model formula, as follows:

(3) $$\text{TL}={\text{TL}}_{\text{base}}+\frac{\delta^{15}N_{c}‐\delta^{15}N_{b}}{\Delta^{15}N}$$

where TL is the consumer trophic level, TL_base_ is the baseline trophic level, δ^15^N_c_ is the consumer nitrogen isotope ratio, δ^15^N_b_ is the marine primary consumer nitrogen isotope ratio, and Δ^15^N is the trophic fractionation factor. Primary consumers occupied the 2nd TL at the base of the trophic ladder, so the δ^15^N value of zooplankton was considered the baseline in this study.

### 2.6 Food source analysis

In this study, we identified five paralic organisms, including autochthonous primary producers (phytoplankton and microphytobenthos) and allochthonous food sources (macroalgae, cordgrass, and organic matter from the Yellow River (YROM)), as the primary energy providers for local paralic food webs and the potential primary food sources of fish species. We analyzed the contributions of each potential food source based on δ^13^C using SIAR and then drew block diagrams illustrating the results. According to Wang et al. (2018), estuarine organic matter is predominately from autochthonous sources, and the estimated autochthonous organic carbon is approximately 58%–82% of total organic carbon. Therefore, POM with a diameter between 20 and 200 μm was deemed representative of estuarine–marine phytoplankton and the associated isotope values were used in food source analysis. Surface SOM (<5 mm) excluded inorganic carbon and was represented by microphytobenthos in local research areas. Enteromorpha and spartina were represented by macroalgae and cordgrass in the subtidal and intertidal zone, respectively. Since the diluted water directly influenced Biotope‐H and Biotope‐C more than Biotope‐B, Biotope‐D, and Biotope‐S, YROM in Biotope‐H and Biotope‐C was included in the food source analysis, while that in Biotope‐B, Biotope‐D, and Biotope‐S was not. The results from previous research indicated that there was no significant difference between YROM and primary terrestrial vegetation in the Yellow River Delta (Qu et al., 2019), so YROM was suitable for representing delta vegetation in a local finite area. Consequently, five potential food sources were identified in Biotope‐H and Biotope‐C, corresponding to a 20% average contribution for fish species, while four potential food sources corresponding to a 25% average contribution were identified in Biotope‐B, Biotope‐D, and Biotope‐S. The contribution of each potential carbon source to fish communities was estimated using SIAR (Jackson et al., 2009; Jackson et al., 2011).

## 3 RESULTS

### 3.1 Variation in δ^13^C and δ^15^N

Seventeen common fish species involving 168 specimens were collected from the five local research biotopes using a consistent sampling method for δ^13^C and δ^15^N analysis (biomass proportions are shown in Table S1). The average δ^13^C values for each species in the five survey areas are shown in Table 1. Biotope‐H was located at the subtidal zone of the northern Yellow River estuary and was affected most by the abundant diluted water from the Yellow River. In our study, δ^13^C values for fish in this biotope had the broadest range, from −22.17‰ to −16.94‰ with an average of −19.52‰. Biotope‐C was located at the subtidal zone of the southern Yellow River estuary, where the effect of diluted water was weaker than the northern area because of silting at the southern river mouth. The δ^13^C values for fish here ranged from −21.50‰ to −17.48‰ with an average of −19.63‰. Biotope‐D, which was located further north of the Yellow River estuary, approaching open water, had less of a diluted water effect. The δ^13^C values for fish here ranged from −23.30‰ to −20.00‰, and the average was −21.40‰. Biotope‐B, located at the edge of the intertidal zone northwest of the Yellow River estuary, was strongly influenced by local diluted water from river branching rather than the Yellow River mainstream. The δ^13^C values for fish here ranged from −19.80‰ to −17.87‰, and the average was −18.87‰. For Biotope‐S, the δ^13^C values for fish ranged from −21.56‰ to −18.68‰ with an average of −20.02‰.

**TABLE 1:** δ^13^C (mean values ± *SD*, *n* = 3) of 17 fish species in five survey areas in 2017 and 2018

No.	Species	B: δ^13^C (‰)	D: δ^13^C (‰)	H: δ^13^C (‰)	C: δ^13^C (‰)	S: δ^13^C (‰)
1	*Argyrosomus argentatus*	−18.02 ± 0.03	−20.70 ± 0.78	−19.31 ± 0.32	−18.27 ± 0.72	−20.87 ± 0.24
2	*Konosirus punctatus*	−18.70 ± 0.06	−21.33 ± 0.64	−19.90 ± 0.13	−19.36 ± 0.06	−21.09 ± 0.03
3	*Cynoglossus semilaevis*	−19.01 ± 0.30	−20.60 ± 0.78	−19.02 ± 0.38	−19.71 ± 0.41	−19.38 ± 0.26
4	*Thryssa kammalensis*	−19.19 ± 0.26	−20.80 ± 0.70	−22.01 ± 0.24	−20.88 ± 0.55	−20.34 ± 1.06
5	*Amblychaeturichthys hexanema*	−19.70 ± 0.07	−20.57 ± 0.81	−20.42 ± 0.43	−19.52 ± 0.70	−19.11 ± 0.00
6	*Sardinella zunasi*	−19.14 ± 0.12	−21.77 ± 0.90	−20.20 ± 0.30	−19.69 ± 0.54	−20.95 ± 0.32
7	*Platycephalus indicus*	−18.49 ± 0.07	−22.97 ± 0.21	−19.20 ± 0.09	−19.66 ± 0.07	−19.11 ± 0.71
8	*Synechogobius hasta*	−18.35 ± 0.31	−20.87 ± 0.72	−20.14 ± 0.34	−19.25 ± 0.23	—
9	*Triaenopogon barbatus*	−19.38 ± 0.50	−21.27 ± 0.67	—	—	—
10	*Thryssa mystax*	−19.29 ± 0.51	—	−20.16 ± 0.82	−20.15 ± 0.29	—
11	*Cynoglossus joyneri*	−18.28 ± 0.36	—	−17.60 ± 0.81	—	—
12	*Enedrias fangi*	—	−23.10 ± 0.20	—	—	—
13	*Sillago japonica*	—	—	−18.99 ± 0.48	—	—
14	*Eupleurogrammus muticus*	—	—	−19.58 ± 0.04	—	—
15	*Odontamblyopus rubicundus*	—	—	−18.28 ± 0.01	−19.79 ± 0.01	—
16	*Setipinna tenuifilis*	—	—	−18.44 ± 0.37	−19.72 ± 1.27	−19.39 ± 0.22
17	*Pampus echinogaster*	—	—	−19.61 ± 0.30	—	−19.93 ± 0.19

The average δ^15^N values are shown in Table 2. Similar to δ^13^C values, the δ^15^N range for fish in Biotope‐H was the broadest, from 7.05‰ to 14.30‰ with an average of 12.08‰. The δ^15^N for fish in Biotope‐B ranged from 10.62‰ to 13.16‰ with an average of 11.97‰. The δ^15^N for fish in Biotope‐D ranged from 10.08‰ to 13.12‰ with an average of 11.57‰. The δ^15^N for fish in Biotope‐C ranged from 10.20‰ to 15.28‰ with an average of 12.57‰, and the δ^15^N for fish in Biotope‐S ranged from 11.89‰ to 17.09‰ with an average of 14.80‰. The δ^15^N data for Biotope‐S were significantly higher than all other biotopes (*p* < 0.01, Table 3, Table S2), while the δ^15^N data for Biotope‐C were significantly higher than those for Biotope‐B and Biotope‐D (*p* < 0.05, Table 3, Table S2).

**TABLE 2:** δ^15^N (mean values ± *SD*, *n* = 3) of 17 fish species in five survey areas in 2017 and 2018

No.	Species	B: δ^15^N (‰)	D: δ^15^N (‰)	H: δ^15^N (‰)	C: δ^15^N (‰)	S: δ^15^N (‰)
1	*Argyrosomus argentatus*	12.96 ± 0.04	11.15 ± 0.82	12.70 ± 1.21	12.58 ± 0.57	15.36 ± 0.77
2	*Konosirus punctatus*	11.24 ± 0.22	11.76 ± 0.89	10.97 ± 0.53	10.38 ± 0.16	11.93 ± 0.07
3	*Cynoglossus semilaevis*	11.31 ± 0.60	10.84 ± 0.90	12.16 ± 0.48	12.36 ± 0.30	13.58 ± 0.06
4	*Thryssa kammalensis*	11.80 ± 0.08	11.27 ± 0.85	12.57 ± 0.06	13.66 ± 0.31	14.94 ± 0.14
5	*Amblychaeturichthys hexanema*	11.62 ± 0.23	10.75 ± 0.94	12.18 ± 0.10	12.39 ± 0.25	15.75 ± 0.17
6	*Sardinella zunasi*	13.01 ± 0.03	11.91 ± 0.84	7.89 ± 0.80	11.46 ± 0.97	15.89 ± 0.09
7	*Platycephalus indicus*	12.30 ± 0.58	12.07 ± 0.56	13.48 ± 0.24	13.57 ± 0.30	14.09 ± 0.03
8	*Synechogobius hasta*	10.98 ± 0.07	11.47 ± 0.51	13.52 ± 0.01	12.70 ± 0.58	—
9	*Triaenopogon barbatus*	11.91 ± 0.06	11.53 ± 0.82	—	—	—
10	*Thryssa mystax*	11.52 ± 0.26	—	11.52 ± 0.17	11.95 ± 0.25	—
11	*Cynoglossus joyneri*	13.03 ± 0.12	—	13.52 ± 0.45	—	—
12	*Enedrias fangi*	—	12.99 ± 0.11	—	—	—
13	*Sillago japonica*	—	—	9.79 ± 0.56	—	—
14	*Eupleurogrammus muticus*	—	—	11.82 ± 0.83	—	—
15	*Odontamblyopus rubicundus*	—	—	12.40 ± 0.67	12.26 ± 0.03	—
16	*Setipinna tenuifilis*	—	—	13.85 ± 0.41	14.90 ± 0.41	14.69 ± 0.16
17	*Pampus echinogaster*	—	—	12.81 ± 0.42	—	16.94 ± 0.15

**TABLE 3:** The output of the one‐way analysis of variance of fish δ^15^N values and TL (trophic level)

		Sum of squares	*df*	Mean Square	*F*	*p*
δ^15^N	Between Groups	185.426	4	46.357	29.477	<0.01
	Within groups	256.344	163	1.573		
	Total	441.770	167			
TL	Between Groups	16.011	4	4.003	29.389	<0.01
	Within groups	22.2	163	0.136		
	Total	38.212	167			

Note

Significant (*p*) values indicate differences among biotopes (B, D, H, C, S) (*p* < 0.01 showed that there were significant differences between the five groups).

### 3.2 Comparing stable isotopic niches using SIBER

**FIGURE 2:**
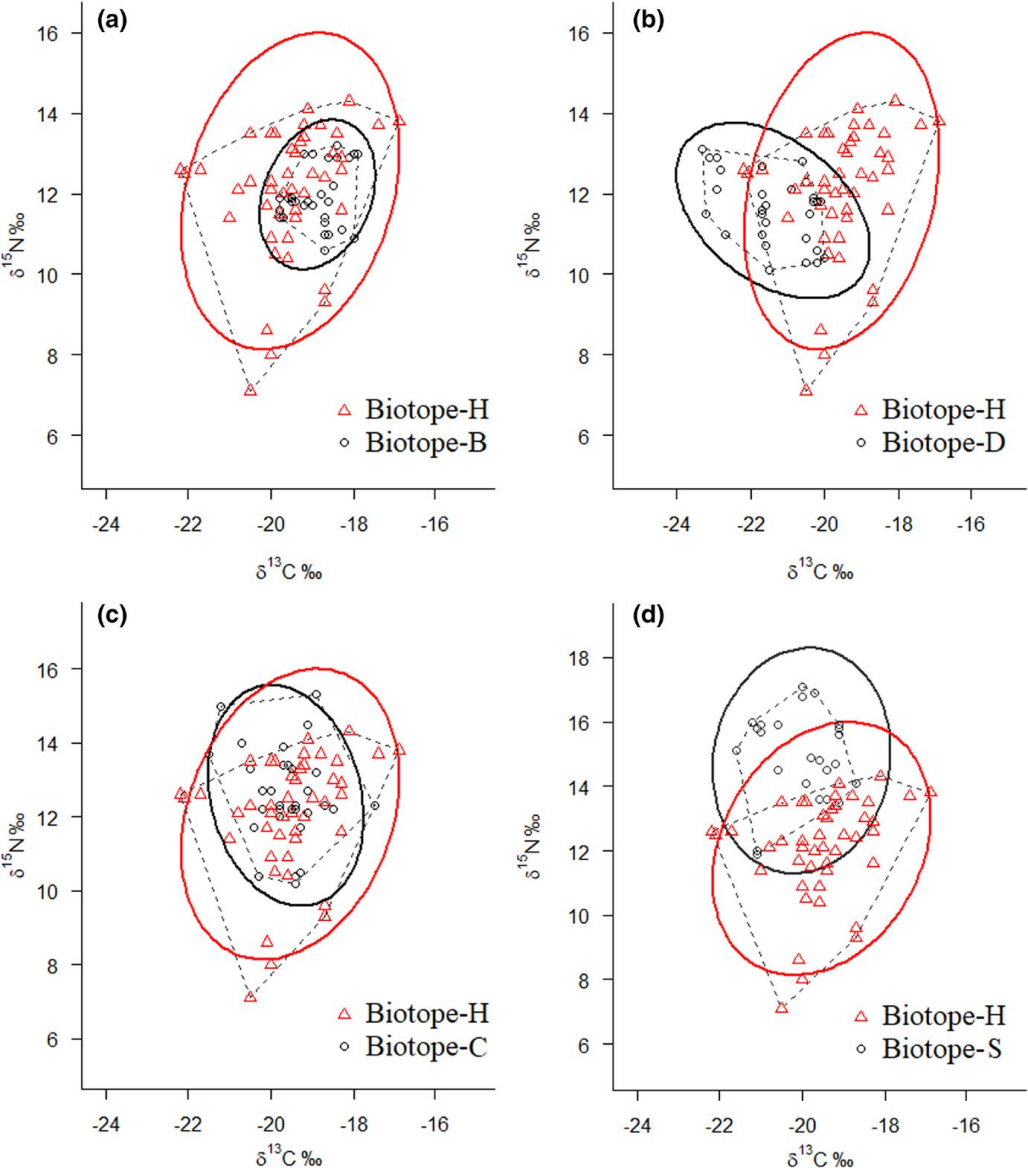
A dot plot of δ^13^C‐δ^15^N with the standard ellipse area (SEAc) illustrating comparisons between Biotope‐H and other biotopes (Biotope‐B, Biotope‐D, Biotope‐C, and Biotope‐S corresponding to inset a, b, c, and d, respectively) using the SIBER package, which included the common species in each of the five sites among the 17 common fish species of the biotopes (biotope‐H was drawn as red triangles, and the other biotopes were drawn as black circles)

Biotope‐H was most influenced by diluted water from the Yellow River, so it was chosen as the object of comparison with the other biotopes (B, D, C, and S), avoiding too complex and mixed‐up dots with their SEA in a single plot using SIBER (Figure 2). The niche width of δ^13^C and δ^15^N for fish is shown in Table 4. In Biotope‐H, the niche width of δ^13^C and δ^15^N was 7.24‰ and 5.24‰, respectively, and both had the widest niche of all selected biotopes (Table 4). Niche widths in Biotope‐B were the narrowest at 1.93 for δ^13^C and 2.53 for δ^15^N. Accordingly, Biotope‐H had the highest SEAc of 5.38 followed by Biotope‐S (4.10) and Biotope‐C (2.98), while Biotope‐B had the lowest SEAc of 1.36. The isotopic niche area of Biotope‐H contained Biotope‐B (Figure 2a), while similarly Biotope‐H almost included Biotope‐C except for one dot (Figure 2c, δ^13^C of −21.18‰, δ^15^N of 14.96‰). The SEAc of Biotope‐D was 2.83, and its δ^13^C value was significantly lower than that of Biotope‐H (*t* test, *p* < 0.01, *n* = 33) (Figure 2b). The SEAc of Biotope‐S was 4.10, and its δ^15^N was significantly higher than that of Biotope‐H (*t* test, *p* < 0.01, *n* = 27) (Figure 2d).

**TABLE 4:** Sampling number (*n*), niche width (δ^13^C and δ^15^N), and SIBER analysis results including the total area (TA), standard Bayesian Ellipse Area (SEA), and ellipse area corrected for small sample size (SEAc) in five different biotopes

Biotopes	*n*	δ^13^C niche width	δ^15^N niche width	TA	SEA	SEAc
B	33	1.93	2.53	3.68	1.32	1.36
D	30	3.30	3.04	7.38	2.73	2.83
H	45	5.24	7.24	20.27	5.26	5.38
C	33	4.02	5.08	13.36	2.89	2.98
S	27	2.88	5.21	9.35	3.94	4.10

### 3.3 Trophic levels

**FIGURE 3:**
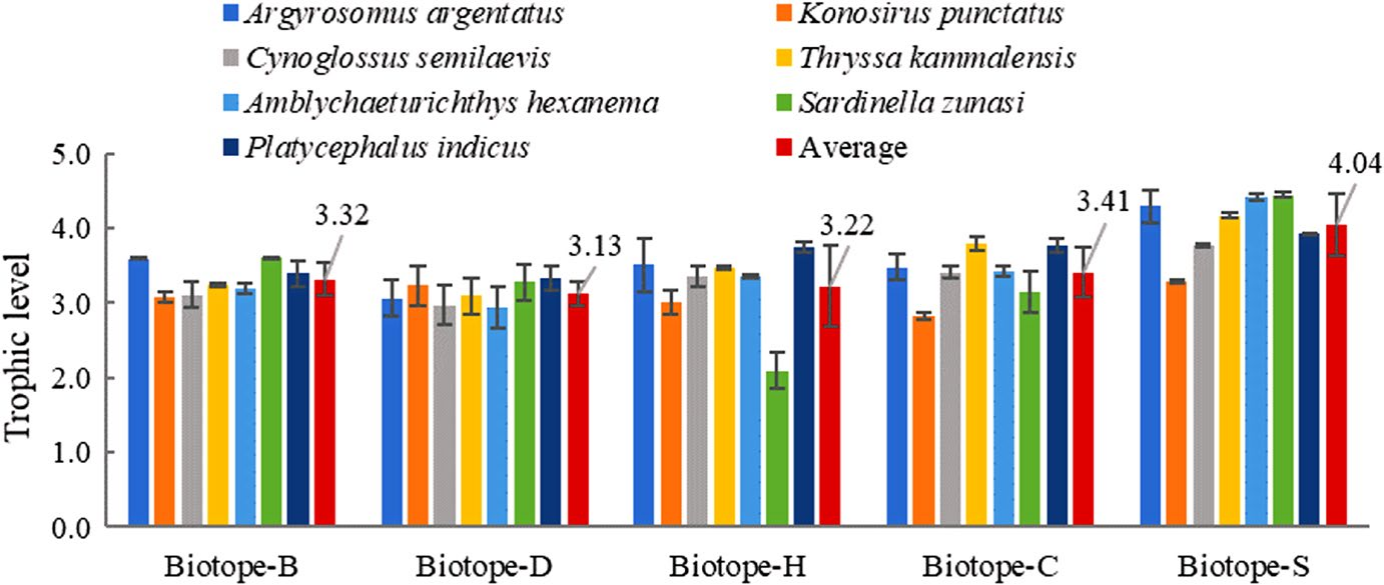
TLs of the seven common fish species (average labeled) in the five biotopes

As seven common fish species (*Argyrosomus argentatus*, *Amblychaeturichthys hexanema*, *Cynoglossus semilaevis*, *Thryssa kammalensis*, *Konosirus punctatus*, *Platycephalus indicus*, and *Sardinella zunasi*) appeared in all five biotopes, they were chosen for TL and food source comparisons in this study. The TLs of these seven species in each biotope were calculated using a unique baseline. Figure 3 shows the average TL of the seven representative fishes in each biotope. Agreeing with δ^15^N data, the highest average TL of 4.0 was found for Biotope‐S, while the lowest average of 3.1 was found for Biotope‐D. The TL of Biotope‐S was significantly higher than any other biotope (*p* < 0.01, Table 3, Table S3). Biotope‐H had the highest standard deviation (0.5), while Biotope‐D had the lowest standard deviation (0.2). For single species, the highest TL was 4.5 (*S. zunasi*) in Biotope‐S, while the lowest TL was 2.1 (also *S. zunasi*) in Biotope‐H.

### 3.4 Food source analysis

**FIGURE 4:**
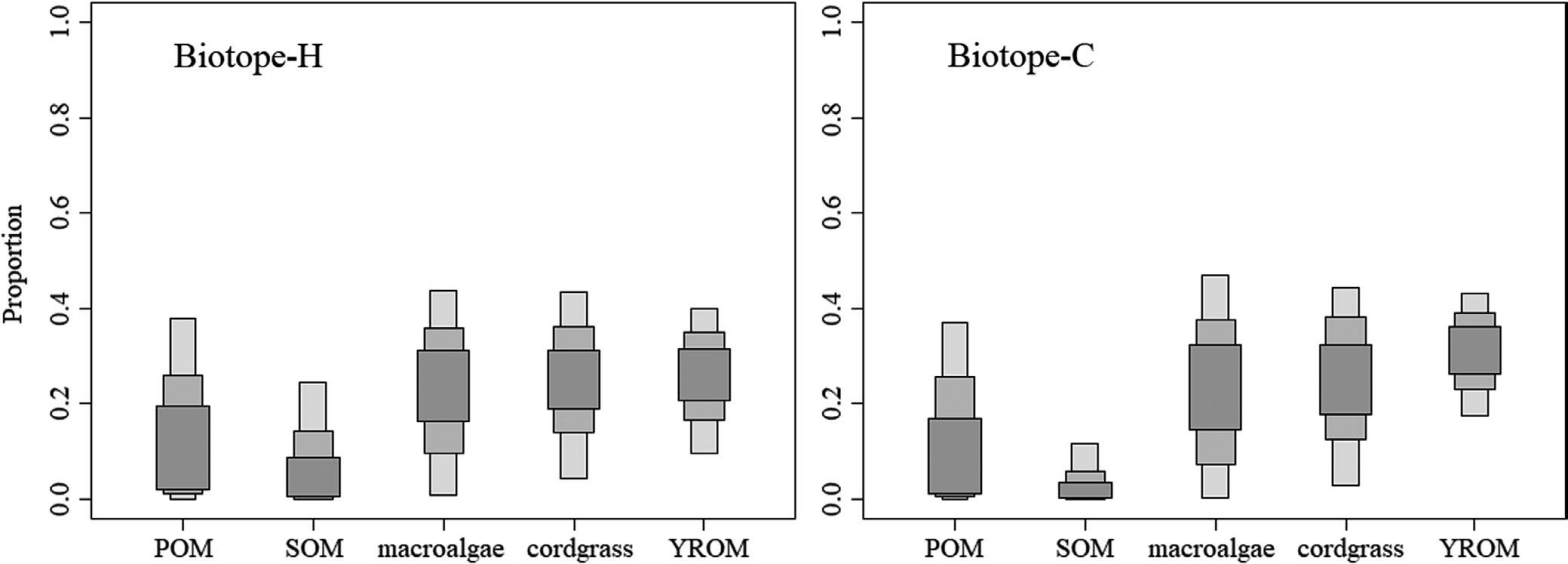
Relative contribution of the five potential food sources to the diet of fish communities in Biotope‐H and Biotope‐C using SIAR. Gray‐shaded areas represent 95%, 75%, and 50% confidence intervals (food source containing POM = suspended particulate organic matter, SOM = sedimental organic matter, macroalgae, cordgrass, and YROM = Yellow River organic matter)

As shown in Figure 4, the contribution of the five potential food sources showed a similar distribution tendency in Biotope‐H and Biotope‐C (Table S4). Allochthonous food sources (macroalgae, cordgrass, and YROM) showed higher proportional contributions to local fish communities than autochthonous sources (POM and SOM) (Figure 4). The 95% confidence intervals (CIs) were 0.01–0.44 for macroalgae, 0.04–0.43 for cordgrass, and 0.10–0.40 for YROM, and 0–0.37 for POM and 0–0.24 for SOM in Biotope‐H. The 95% CIs were 0–0.47 for macroalgae, 0.03–0.44 for cordgrass, and 0.17–0.43 for YROM, and 0–0.37 for POM and 0–0.12 for SOM in Biotope‐C. The 95% CIs for macroalgae showed the widest distribution, which was 0.43 in Biotope‐H and 0.47 in Biotope‐C, respectively. YROM demonstrated less variation in the confidence interval, which was 0.30 in Biotope‐H and 0.16 in Biotope‐C. Conversely, SOM showed the lowest contribution, with a 95% CI of 0–0.24 in Biotope‐H and 0–0.12 in Biotope‐C, respectively. For single species, both macroalgae and cordgrass contributed relatively more to *A. argentatus* (mean = 0.33 and 0.30 in Biotope‐H; 0.32 and 0.33 in Biotope‐C), while YROM contributed a 0.37 mean proportion for *T. kammalensis* in Biotope‐H. More detailed food source contribution data for fish species are shown in Tables S4 and S5.

**FIGURE 5:**
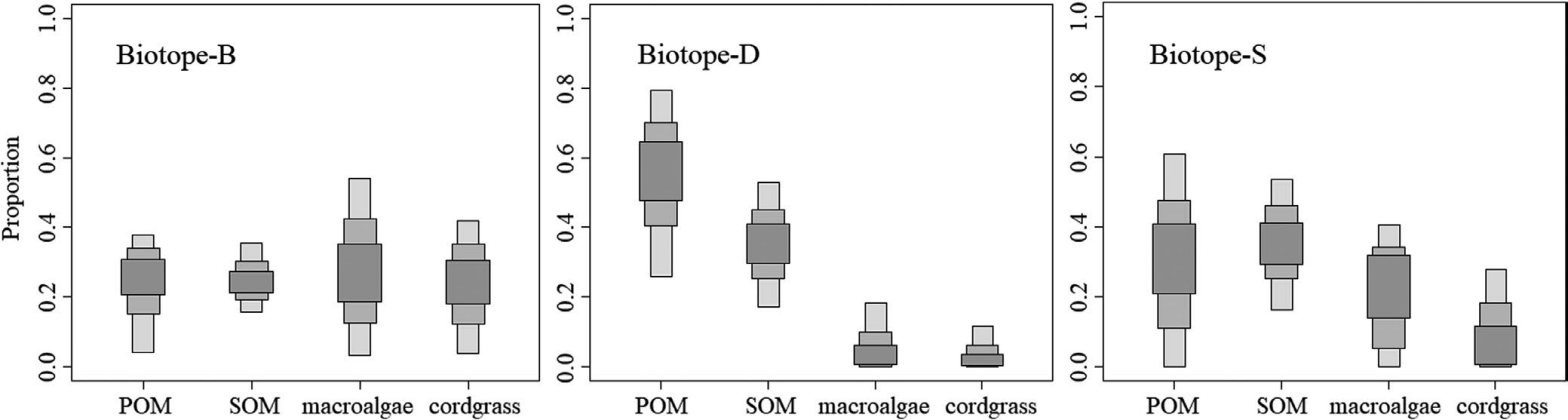
Relative contribution of the five potential food sources to the diet of fish communities in Biotope‐B, Biotope‐D, and Biotope‐S using SIAR. Gray‐shaded areas represent 95%, 75%, and 50% confidence intervals (food source containing POM = suspended particulate organic matter, SOM = sedimental organic matter, macroalgae, and cordgrass)

In Biotope‐D, which was the farthest offshore, POM showed a significantly higher food source contribution, and its 95% CI was higher (0.26–0.79) than any other potential food sources (Figure 5, Table S4), and for single species, the highest mean contribution of POM was to *K. punctatus* (0.76) in Biotope‐D (Table S5). The 95% CI for SOM was also higher (0.17–0.53) than for macroalgae and cordgrass, which showed extremely low 95% CIs of 0–0.18 and 0–0.11, respectively. In Biotope‐S, POM and SOM were also considered to be the primary food sources based on contribution results, which accounted for 0–0.61, but with high distribution indeterminacy (0.61), and 0.16–0.54 for 95% CIs, respectively. In Biotope‐B, the contributions of each food source were in relative equilibrium, while for single species, cordgrass showed a 0.48 contribution to *A. argentatus* (Table S5).

## 4 DISCUSSION

Increasing numbers of studies have shown that coastal and estuarine ecological connectivity plays an essential role in ecosystem conservation and restoration (Du et al., 2015). In this study, we focused on the variation in stable isotope space. Two‐year sampling was selected for the purpose of complementarity to ensure a more complete collection of common species in the study area. Before the analysis of regional differences, no significant difference (*t* test: δ^13^C, *p* > 0.05; δ^15^N, *p* > 0.05) between the two sampling periods (2017 and 2018) was verified to confirm that the difference was not the result of temporal changes. The stable isotopic niche results in this study indicated that the SEAc (5.38) and total area (TA, 20.27) of estuarine Biotope‐H covered the majority of the other research areas (Figure 2, Table 4). A comparative analysis of stable isotopic niches was useful for detecting patterns in trophic structure and identifying differences or similarities in trophic organization related to environmental conditions (Abrantes et al., 2014).

### 4.1 δ^13^C niche variation

The δ^13^C data in our study indicated that all fish species mainly corresponded to benthic diatoms, macroalgae, and estuarine–marine phytoplankton (Cloern et al., 2002), which occupy their marine isotopic niches (δ^13^C: range from −24.07‰ to −16.82‰) (Newsome et al., 2007). Beyond macroalgae, our analysis of the food source contribution indicated that YROM and cordgrass were major allochthonous energy sources in estuarine areas directly affected by Yellow River‐diluted water, while local autochthonous primary producers (phytoplankton and microphytobenthos) demonstrated a low contribution in those specific areas (Figure 4). Phytoplankton produce new particles that drive the biological carbon pump, contributing to the global carbon cycle in the ocean, which plays a disproportionately important role in the global climate on a range of time scales (Bolaños et al., 2020; Moreau et al., 2020). However, it is susceptible to environmental conditions relative to other primary producers, especially the variable environment of estuaries. The bloom and extinction of phytoplankton is driven by physical, chemical, and biological seasonality (Bolaños et al., 2020). Hydrology and dissolved nutrients have been widely identified as the main drivers of phytoplankton dynamics in estuarine ecosystems (Tao et al., 2020). Our investigation indicated that a high concentration of chlorophyll‐a, a representative of phytoplankton (Moreau et al., 2020), had not shown up in estuarine areas with a direct diluted water influence (Appendix 1). This is consistent with the results of Ding et al. (2020). The contribution of microphytobenthic primary producers also was low, probably due to their low biomass resulting from light limitation (Haro et al., 2019; Wang et al., 2017). On the other hand, besides macroalgae, allochthonous energy sources were identified as the main food sources supporting the estuarine food web. In a previous study, most of the riverine organic carbon originated from delta vegetation debris (Phragmites, Suaeda, and Tamarisk) in particulate form (Wang et al., 2018). Suspended particulate matter acts as the main carrier of organic matter, providing energy to the estuarine food web from upstream carrying, which plays an important role in the conditioning of productivity and ecosystem functions in estuaries (Li et al., 2020). As a representative of cordgrass in the intertidal zone, spartina provided a considerable food contribution proportion for estuarine fish communities in this study. However, it was recognized as the main invasive plant in the Yellow River estuarine area, so its contribution to the local estuarine food web is still controversial (Chen et al., 2020). *Spartina alterniflora* was first introduced to the coastal wetlands of China from the United States in 1979 for the purpose of ecological restoration. From 1985 to 2015, it continued to spread across the coast of mainland China as a typical invasive species (Meng et al., 2020).

Areas away from the Yellow River estuary show different food contribution characteristics compared with the estuarine area. As indicated by the food source contribution results, autochthonous benthic and pelagic producers (microphytobenthos and phytoplankton) dominated carbon input into the food web in Biotope‐S and Biotope‐D, which conformed to the normal characteristics of an intertidal ecosystem like the Wadden Sea (Christianen et al., 2017). Microphytobenthos form extensive biofilms on the sediment surface conducive to its stabilization. They are not easily disturbed and thus provide a more stable food source for local consumers (Hart & Lovvorn, 2003; Miyatake et al., 2014). In contrast, phytoplankton are more vulnerable to influence from environmental conditions (Armbrecht et al., 2015), while providing an unstable food source according to the more discrete confidence interval of contribution (Figure 5).

Caut et al. (2009) reported that the discrimination factor of δ^13^C for muscle differed significantly among birds, fishes, and mammals, but it did not differ significantly for plasma and liver. For the whole body, it differed significantly between invertebrates and fishes. The overall mean estimate was 0.75‰ (*n* = 290) for these four groups of organisms. This value should be subtracted when comparing consumers with their potential food sources. However, Caut et al. (2009) also reported that the discrimination factors of δ^13^C remain problematic and advised caution in the use of a single discrimination factor in isotopic models. Under the current circumstances, we were not able to obtain a precise fractionation factor in the research area, so we used zero as the fractionation factor to avoid incurring greater uncertainty and because of cost and practicality issues. However, if possible, researchers should try to determine the diet‐dependent discrimination factor as a tool for obtaining more accurate results when using isotope models (Caut et al., 2010).

### 4.2 δ^15^N niche variation

The trophic level (TL), which ranged from 2.1 to 4.5 using a unique baseline (Figure 3), indicated that the fish species in our study system covered a distance of 2.4, which differed from the general trophic pattern of fish communities in Chinese coastal waters, such as the range from 3.0 to 4.1 for the Changjiang Estuary at the junction of the Yellow Sea and the East China Sea (Chang et al., 2014), from 3.1 to 3.6 in the coastal waters of the Yellow Sea (Feng et al., 2014), and from 2.9 to 3.9 at the junction of the East China Sea and South China Sea (Du et al., 2015). Their trophic level was also wider than that in the western Mediterranean (range from 2.9 to 4.0, Valls et al., 2014), but lower than that in the Gulf of Maine (range from 3.7 to 5.2, Schartup et al., 2019). From the above comparisons, the variation in TL in our research area (2.4) was much wider than in other similar coastal areas. Intriguingly, this variation was reflected not only in single species, but also significantly in the biotopes according to the results of this study (Table 3, Table S3). Our results demonstrated that the average fish TL was 3.3 in Biotope‐B, 3.1 in Biotope‐B, 3.2 in Biotope‐H, 3.4 in Biotope‐C, and 4.0 in Biotope‐S, giving the trend of S > C > B > H > D. This tendency showed that the average TL was significantly higher in Laizhou Bay than any other biotope and decreased from the near shore biotope to the far shore, which meant significant variation in our original δ^15^N data. It was an abnormal phenomenon that similar species had such a significantly different δ^15^N characteristic between connected biotopes.

δ^15^N can also be used to indicate scenopoetic dimensions, such as marine‐terrestrial (Lange et al., 2018) and eutrophication (Gooddy et al., 2016). Generally, the difference in habitat conditions is mainly related to the scenopoetic dimensions where a high δ^15^N value indicates a marine characteristic while a low value indicate a terrestrial characteristic; a high value also indicates a eutrophic area while a low value indicates a pristine area (Newsome et al., 2007). The environmental condition of Laizhou Bay is closer to a mainland, and it belongs more to a terrestrial rather than a marine characteristic with its lower δ^15^N value, and thus, the results in our study did not obey the marine‐terrestrial pattern. We also suspected that local aquaculture activities might lead to a high δ^15^N value from wild marine lives by releasing organic bait based on similar conditions in the aquaculture water in Jiaozhou Bay, off the coast of China (Feng et al., 2014). Therefore, a field survey was conducted in May 2020 and scallop culture was identified as the main local aquaculture with no release of anthropogenic organic bait. After excluding the above conditions, we needed a more reasonable theory to explain the abnormal phenomenon in our study area.

After the above analysis, we turned our attention to probing into the δ^15^N variation based on the food source contribution. Whether in a high δ^15^N (Biotope‐S) or low δ^15^N (Biotope‐D) area, the autochthonous food source demonstrated a relatively high contribution to the local fish communities, which implied that it is probably due to the influence of primary producers at the base of the food web (Oakes et al., 2010). However, primary producers seldom directly provide energy to high‐TL predators (Warne et al., 2010), so it does not adequately explain the δ^15^N variation in different biotopes.

**FIGURE 6:**
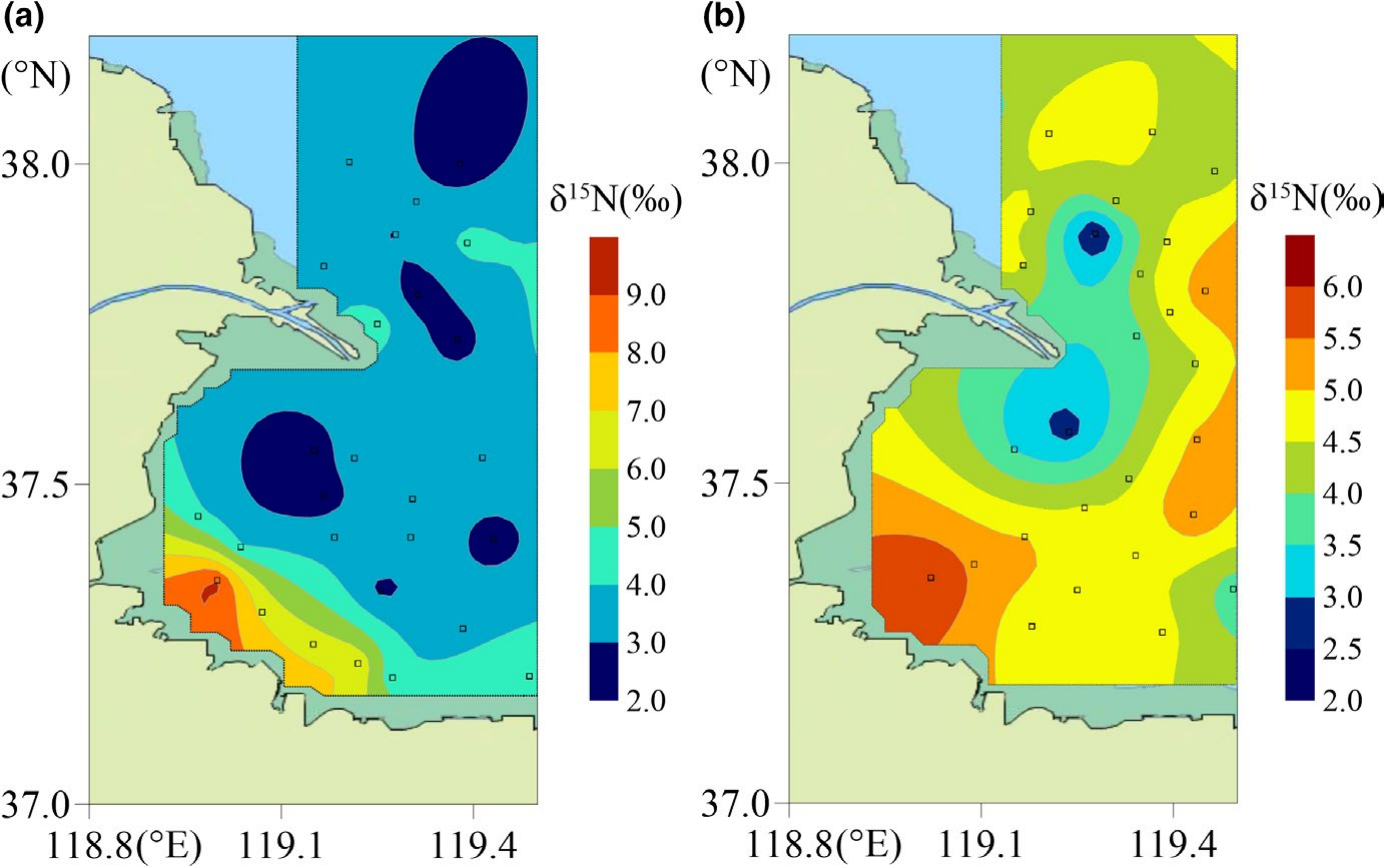
The distribution of δ^15^N in organic sediment in August 2017 (a, 26 locations of sample sites are indicated by squares) and May 2020 (b, 27 locations of sample sites are indicated by squares) mapped using Surfer 13.0 with the Kriging Gridding method

The inorganic nitrogen assimilation process is a key driver from primary producers in the marine nitrogen cycle (Hetherington et al., 2017). Due to the high and stable contribution for fishes, SOM was considered a primary food source in Biotope‐S. We further investigated its δ^15^N distribution (Figure 6) and found a strong link between the high δ^15^N of fish and the distribution of dissolved inorganic nitrogen (DIN) (Appendix 3). Nitrogen‐fixing microorganisms, such as nitrospinae, were also significantly enriched in ^15^N under conditions of a high inorganic nitrogen concentration (Kitzinger et al., 2020). Therefore, the high δ^15^N value of SOM was most probably caused by microorganisms and other primary producers assimilating high‐concentration DIN, which was likely the reason for the eutrophic pattern of δ^15^N (Newsome et al., 2007). The major primary consumers, zooplankton, tended to respond to this variation (Schmidt et al., 2003). This characteristic would translate to high‐TL consumers like fish communities via marine food chains leading to biases in the statistical process (Auerswald et al., 2010; Layman et al., 2012).

Many previous studies found that their application was further complicated by potential shifts in baseline δ^15^N for many specific ecological processes, such as migration of marine nekton like bluefin tuna and swordfish (Schartup et al., 2019), significant taxonomic variation in the composition of primary producers at the base of the food webs (Ramshaw et al., 2017), and the supply of DIN sources (Kitzinger et al., 2020). The δ^15^N characteristic of primary producers may vary by as much as 10‰ over a spatial and temporal scale (McMahon et al., 2015). Therefore, identifying an appropriate baseline requires not only considering migratory predators but also paying more attention to the local primary producers that can determine the baseline of marine trophic structures more directly.

If our conclusion is right that the local high δ^15^N of fishes originated from the high concentration of DIN in Biotope‐S, a trophic model relying on the δ^15^N characteristic would be further complicated by potential shifts in the baseline due to variations in the δ^15^N characteristic in primary producers (Gutiérrez‐Rodríguez et al., 2014). Regional δ^15^N diversity in primary producers should be considered not only between broad oceans on a large spatial scale (Hetherington et al., 2017; Schmidt et al., 2003), but also among adjacent coastal waters with high DIN variation, such as the estuary. Though the research areas in our study were not significantly isolated, the bias of TLs in fish communities still emerged between biotopes, which indicates the diversity of matter and energy flows (Palmer & Ruhi, 2019).

## 5 CONCLUSION

Stable isotopic niche results indicated that organisms inhabiting estuarine ecosystems showed the compatibility of the communities of most study biotopes. YROM and cordgrass were considered to be the major allochthonous energy sources in estuarine areas directly affected by Yellow River‐diluted water, while phytoplankton and microphytobenthos demonstrated a low contribution as local autochthonous primary producers. Areas away from the Yellow River estuary showed different food contribution characteristics compared with estuarine areas. As indicated by the food source contribution results, autochthonous benthic and pelagic producers (microphytobenthos and phytoplankton) dominated carbon input into food webs. Our results showed that the significant variation in the fish δ^15^N characteristic presented within estuarine adjacent regions (less than 2 degrees latitude) led to significant variation in TLs in the same fish species, using a unique baseline. Although the research areas in our study were not significantly isolated, the bias of TLs in fish communities still emerged between biotopes. This indicates the diversity of matter and energy flows. Regional δ^15^N diversity in primary producers should be considered not only between broad oceans on a large spatial scale, but also among adjacent coastal waters with high DIN variation. These results offer a new perspective on trophic relationships and provide the first detailed data for enhancing our understanding of the variations among fish communities in estuarine ecosystems.

## CONFLICT OF INTEREST

The authors have no conflict of interests to declare.

## AUTHOR CONTRIBUTIONS

**Pei Qu:** Conceptualization (lead); Data curation (lead); Formal analysis (lead); Funding acquisition (equal); Investigation (lead); Methodology (lead); Project administration (supporting); Resources (supporting); Software (lead); Validation (lead); Writing‐original draft (lead); Writing‐review & editing (lead). **Min Pang:** Conceptualization (supporting); Data curation (supporting); Formal analysis (equal); Methodology (equal); Software (supporting); Validation (supporting); Writing‐original draft (supporting); Writing‐review & editing (equal). **Fangyuan Qu:** Conceptualization (supporting); Formal analysis (supporting); Writing‐original draft (supporting); Writing‐review & editing (supporting). **Zhao Li:** Conceptualization (supporting); Data curation (supporting). **Meng Xiao:** Conceptualization (supporting); Formal analysis (supporting); Investigation (supporting). **Zhaohui Zhang:** Conceptualization (supporting); Project administration (lead); Resources (lead); Supervision (lead).

## ETHICAL APPROVAL

The authors adhered to all standards for the ethical conduct of research.

### OPEN RESEARCH BADGES

This article has earned an Open Materials Badge for making publicly available the components of the research methodology needed to reproduce the reported procedure and analysis. All materials are available at https://doi.org/10.6084/m9.figshare.14702151.v1.

## Supporting information

Appendix S1

## Data Availability

Supporting information is archived in the “Figshare” data repository and is available at the online resource https://doi.org/10.6084/m9.figshare.14702151.v1.
