# Supplementary material for: Natural distinction of carbon and nitrogen isotopic niches in common fish species across marine biotopes in the Yellow River estuary
Source: Ecol Evol. 2021 Jul 9;11(16):11077–89. doi: 10.1002/ece3.7895 (PMC8366839; doi:10.1002/ece3.7895)

Appendix 1. Chlorophyll-a distribution off Yellow River estuary and adjacent sea areas

Method

In the research area, chlorophyll-a (Chl-a) was surveyed in thirty-two sites. Surface Chl-a data were collected by SeaBird 25plus sealogger CTD sampler equipped fluorometer, temperature, salinity and depth sensor during august 2015 and May 2016. The calibration process used the fluorometric method described by Parsons et al. (1984). Samples were filtered onto Whatman GF/F filters (≈ 0.7 μm) and extracted with 10 ml 90% acetone for 14 to 24 h at 4 ℃ in the dark. The fluorescence was measured before and after acidification with a Turner Designs Model 10-AU fluorometer. The extracted Chl-a concentrations were used to calibrate the fluorescence sensor with linear regression (Fu et al., 2018).

Result and discussion

Fig. S7 showed the maximum value of Chl-a concentrations was 11.01μg/L in August 2015 and 7.97μg/L in May 2016, which appeared both in the southern research area. Our investigation indicated that the high concentration of chlorophyll-a, as a representative of phytoplankton (Moreau et al., 2020), had not showed up in the estuarine areas with direct diluted water influence, which consistent with the results of Ding et al., 2020.

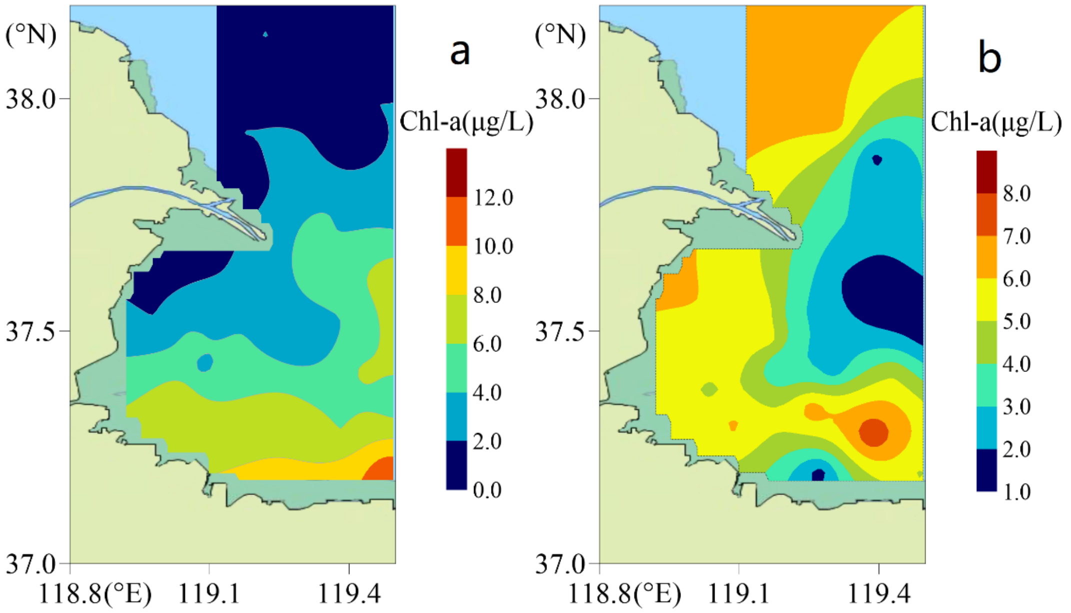

Figure S1 The Chl-a distribution in August 2015 (a) and May 2016 (b) using Surfer 13.0 with Kriging Griding method (Totally 31 sampling sites in each cruise).

Reference

Ding, X., Guo, X., Zhang, C., Yao, X., Liu, S., Shi, J., … Gao, H. (2020). Water conservancy project on the Yellow River modifies the seasonal variation of Chlorophyll-a in the Bohai Sea. Chemosphere, 254, 126846. https://doi.org/10.1016/j.chemosphere.2020.126846

Fu, M., Sun, P., Wang, Z., Wei, Q., Qu, P., & Zhang, X. (2018). Structure, characteristics and possible formation mechanisms of the subsurface chlorophyll maximum in the Yellow Sea Cold Water Mass. Continental Shelf Research, 165(July), 93–105. https://doi.org/10.1016/j.csr.2018.07.007

Moreau, S., Boyd, P. W., & Strutton, P. G. (2020). Remote assessment of the fate of phytoplankton in the Southern Ocean sea-ice zone. Nature Communications, 11(1). https://doi.org/10.1038/s41467-020-16931-0

Parsons, T.R., Maita, Y., Lalli, C.M., 1984. A Manual of Chemical and Biological Methods for Seawater Analysis. 107–109. Pergamon Press, Oxford (New York/Sydney), pp. 115–122.

Appendix 2. Trophic level revised with new method

Method

The trophic levels were determined based on the nitrogen isotopic fractionation for ^{15}N enrichment through the food chains considering with consumers' ingesting and metabolic process (Caut et al., 2009, 2010). However, Hussey et al. (2014) revealed Δ^{15}N was not a constant, and determined an empirical linear relationship with δ^{15}N of consumer through meta-analysis. To exclude the possible error that caused by calculating process, we also used Hussey's equation recalculated the TL (Hussey et al., 2014; Reum et al., 2015). Following this, the TL analysis also used a scaled Δ^{15}N trophic framework that was a narrowing discrimination with increasing trophic levels, which provided a more accurate representation of species structuring (Reum et al., 2015) with the following equation (1) and the corresponding TL equation (2):

$$\Delta^{15}N = \frac{\beta_0 - \beta_1 \times \delta^{15}N_{TL}}{1-\beta_1} \quad (1)$$

$$TL = \frac{log(\delta^{15}N_{lim} - \delta^{15}N_{base}) - log(\delta^{15}N_{lim} - \delta^{15}N_{TL})}{k} + TL_{base} \quad (2)$$

$$k = -log\left(\frac{\beta_0 - \delta^{15}N_{lim}}{-\delta^{15}N_{lim}}\right) \quad (3)$$

$$\delta^{15}N_{lim} = \frac{-\beta_0}{\beta_1} \quad (4)$$

where $\delta^{15}N_{TL}$ is the δ^{15}N of the consumer. β_0 is 5.92 and β_1 is −0.27, which are the median of the highest posterior density (95% confidence intervals) based on experimental data of scaled trophic framework (Hussey et al., 2014). TL_{base} is the TL of baseline, $\delta^{15}N_{lim}$ is the saturating δ^{15}N limit as TL increases, and *k* is the rate at which δ^{15}N approaches $\delta^{15}N_{lim}$ per TL. Both values of $\delta^{15}N_{lim}$ and *k* are obtained from meta-analysis (Hussey et al., 2014) with the following equations.

In May 2020, we reanalyzed the zooplankton, which was considered a mediator of energy-transfer from primary producers to high-TL predators and tended to respond to variations in food source δ^{15}N values (Schmidt et al., 2003), and then recalculated the TL of fishes using zooplankton δ^{15}N values as the baseline in each biotope (Figure 6).

Result

Compared with the unique baseline, the differences in fish TLs were smaller between each biotope (Figure S2 TL_b). The fish TL in Biotope-C was no longer significantly higher than that in Biotope-B and Biotope-D. Though the fish TL in Biotope-S was

still significantly higher than that in the other biotopes, the gaps decreased from 0.83 to 0.42 with Biotope-B, from 0.95 to 0.69 with Biotope-D, from 0.80 to 0.33 with Biotope-H, and from 0.65 to 0.39 with Biotope-C (Appendix Table S6). However, the bias caused by abnormally high $\delta^{15}N$ still existed in the optimized results. This method demonstrated a method for solving the disparity generated from $\delta^{15}N$ data in spatial distribution, which implied a tendency that the $\delta^{15}N$ variation originated in a more fundamental part of the estuarine food web, such as nitrogen cycling dynamics (Hetherington et al., 2017).

To exclude possible error introduced during the calculating process, we also used Hussey's equation to recalculate the TL (Hussey et al., 2014; Reum et al., 2015). Based on the new equation, there were some changes in the TL results, but they still retained the same trend in terms of the average biotope TL (Figure S2, TL_n). Following new equation, there were some changes in the TL results, but retained the same trend on the average TL of biotopes (Figure S2).

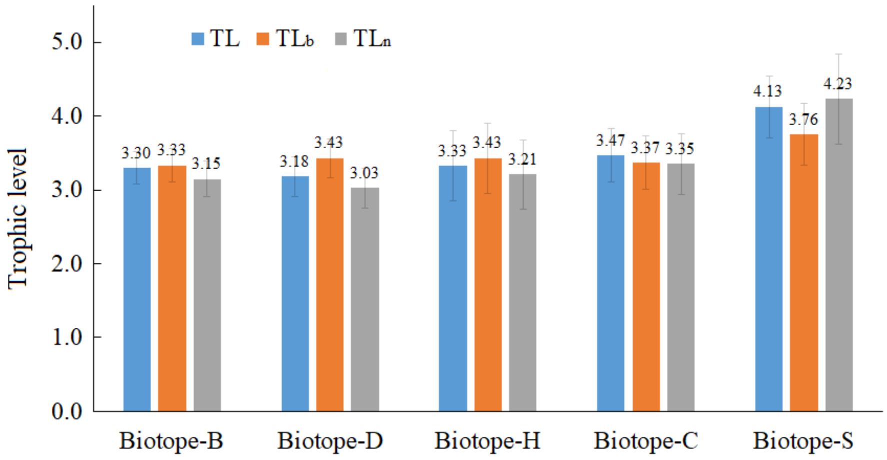

Figure S2. Average TLs of fishes in each biotope using Post's (TL) and Hussey's (TL_n) methods, and revised TLs using a different baseline value ($\delta^{15}N$ of primary consumers) Post's method (TL_b)

Reference

Caut, S., Angulo, E., Courchamp, F., & Figuerola, J. (2010). Trophic experiments to estimate isotope discrimination factors. Journal of Applied Ecology, 47(4), 948–954. https://doi.org/10.1111/j.1365-2664.2010.01832.x

Caut, S., Angulo, E., & Courchamp, F. (2009). Variation in discrimination factors ($\Delta^{15}N$ and $\Delta^{13}C$): The effect of diet isotopic values and applications for diet

reconstruction. Journal of Applied Ecology, 46(2), 443–453. https://doi.org/10.1111/j.1365-2664.2009.01620.x

Hetherington, E. D., Olson, R. J., Drazen, J. C., Lennert-Cody, C. E., Ballance, L. T., Kaufmann, R. S., & Popp, B. N. (2017). Spatial food-web structure in the eastern tropical Pacific Ocean based on compound-specific nitrogen isotope analysis of amino acids. Limnology and Oceanography, 62(2), 541–560.

Hussey, N. E., Macneil, M. A., Mcmeans, B. C., Olin, J. A., Dudley, S. F. J., Cliff, G., … Fisk, A. T. (2014). Rescaling the trophic structure of marine food webs. Ecology Letters, 17(2), 239–250. https://doi.org/10.1111/ele.12226

Reum, J. C. P., Jennings, S., & Hunsicker, M. E. (2015). Implications of scaled δ15N fractionation for community predator-prey body mass ratio estimates in size-structured food webs. Journal of Animal Ecology, 84(6), 1618–1627. https://doi.org/10.1111/1365-2656.12405

Schmidt, K., Atkinson, a., Stubing, D., McClelland, J. W., Montoya, J. P., & Voss, M. (2003). Trophic relationships among Southern Ocean copepods and krill: some uses and limitations of a stable isotope approach. Limnology and Oceanography, 48(1), 277–289.

Appendix 3. Distribution of dissolved inorganic nitrogen (DIN)

Method

In the research area, the dissolved inorganic nitrogen (DIN) was surveyed in twenty sites in May and August, 2016. Water samples for DIN (including nitrite, nitrate, ammonium) were filtered through GF/F filters (≈ 0.7 μm), and then cryopreserved at 4°C. The filtrates were measured after collection with a QuAAtro39 Continuous Flow Analyzer (made by SEAL Ltd., Germany) within one day.

Result and discussion

Due to the high and stable contribution of sediment organic matter (SOM) as a food source in Biotope-S, we further investigated its $\delta^{15}N$ distribution and thereby found the high similarity with high $\delta^{15}N$ of fish and the distribution of dissolved inorganic nitrogen (DIN) (Figure S3). The distribution showed that significant high concentration of DIN was originated from the southwest bottom of Laizhou Bay and gradually decreased to the north (Figure S3 b, c, d, e, f), which corresponded to the high $\delta^{15}N$ value of SOM (Figure S3 a) and fish community (figure 2) in Biotope-S. DIN mainly including nitrate (NO_3^-), nitrite (NO_2^-) and ammonium (NH_4^+) in the ocean (Zhang et al., 2020) can be used by both micro producer and macroalgae. Experimental results proved that the $\delta^{15}N$ value of macroalgae increased with the concentration of DIN in the marine environment (Teichberge et al., 2010). Nitrogen fixing microorganisms, for example nitrospinae, were also significantly enriched in ^{15}N when in the condition of high concentration of inorganic nitrogen (Kitzinger et al., 2020), which were quite possibly the reason of the eutrophic pattern of $\delta^{15}N$ (Newsome et la., 2007). Therefore, the high $\delta^{15}N$ value of SOM was most probable caused by microorganism and other primary producers in the condition of assimilating high-concentration DIN, and then this feature would translate to the high consumers like fish community via marine food chains leading more biases in statistical process (Auerswald et al., 2010; Layman et al., 2012). So, if we intend to solve this issue fundamentally, the baseline should be optimized with comprehensive and integrated analysis in the further research. And this inference will be more specifically verified in our next step study, because it cannot be adequately confirmed just rely on available data to explain the integrated transfer process. Although this inference

related a series of transfer processes from high concentration DIN to primary producers, to primary consumers, and then to the higher consumers like fishes, which were so complicated and contained too many intermediate processes, it would be the most likely cause to interpret this abnormal phenomenon relied on the previous theory (Newsome et al., 2007; Teichberge et al., 2010; Layman et al., 2012; Kitzinger et al., 2020) combining with our results, which reflected highly connected loops of food web.

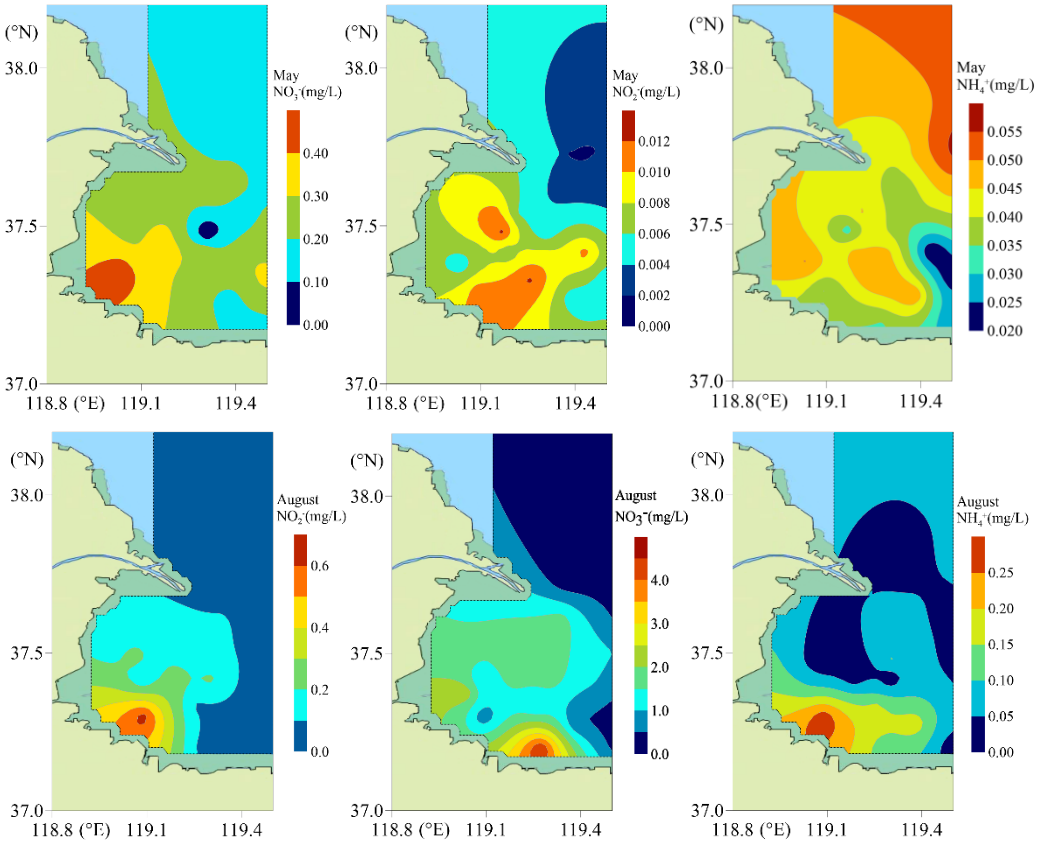

Figure S3. The distribution of sediment organic $\delta^{15}N$ and distribution of seawater DIN (NO_3^-, NO_2^- and NH_4^+) in May and August, 2016 using Surfer 13.0 with Kriging Griding method (Totally 21 sampling sites in each cruise).

Reference

Auerswald, K., Wittmer, M. H. O. M., Zazzo, A., Schäufele, R., & Schnyder, H. (2010). Biases in the analysis of stable isotope discrimination in food webs. Journal of Applied Ecology, 47(4), 936–941. https://doi.org/10.1111/j.1365-2664.2009.01764.x

Kitzinger, K., Marchant, H. K., Bristow, L. A., Herbold, C. W., Padilla, C. C., Kidane, A. T., … Kuypers, M. M. M. (2020). Single cell analyses reveal contrasting life

strategies of the two main nitrifiers in the ocean. Nature Communications, 11(1), 767. https://doi.org/10.1038/s41467-020-14542-3

Layman, C. A., Araujo, M. S., Boucek, R., Hammerschlag-Peyer, C. M., Harrison, E., Jud, Z. R., … Bearhop, S. (2012). Applying stable isotopes to examine food-web structure: An overview of analytical tools. Biological Reviews, 87(3), 545–562. https://doi.org/10.1111/j.1469-185X.2011.00208.x

Newsome, S. D., Rio, Martinez del, C., Bearhop, S., & Phillips, D. L. (2007). A Niche for Isotope Ecology. Frontiers in Ecology and the Environment, 5(8), 429–436. https://doi.org/10.1890/060150.01

Teichberg, M., Fox, S. E., Olsen, Y. S., Valiela, I., Martinetto, P., Iribarne, O., … Tagliapietra, D. (2010). Eutrophication and macroalgal blooms in temperate and tropical coastal waters: Nutrient enrichment experiments with Ulva spp. Global Change Biology, 16(9), 2624–2637. https://doi.org/10.1111/j.1365-2486.2009.02108.x

Zhang, M., Huang, G., Liu, C., Zhang, Y., Chen, Z., & Wang, J. (2020). Distributions and origins of nitrate, nitrite, and ammonium in various aquifers in an urbanized coastal area, south China. Journal of Hydrology, 582(October 2019), 124528. https://doi.org/10.1016/j.jhydrol.2019.124528

Table S1. Average biomass (AB) and quantity (AQ) of fishes, Biomass Proportion (BP) and Quantitative Proportion (QP)

	No.	Species	AB (g/site)	AQ (inds. /site)	BP (%)	QP (%)
Biotope-B	1	*Sardinella zunasi*	3897.7	976.7	38.8	39.5
Biotope-B	2	*Amblychaeturichthys hexanema*	3402.5	596.7	33.9	24.1
Biotope-B	3	*Synechogobius hasta*	1195.4	76.7	11.9	3.1
Biotope-B	4	*Thryssa kammalensis*	407.3	608.3	4.1	24.6
Biotope-B	5	*Cynoglossus semilaevis*	317.3	28.3	3.2	1.1
Biotope-B	6	*Cynoglossus joyneri*	245.8	1.7	2.4	0.1
Biotope-B	7	*Konosirus punctatus*	211.6	45.0	2.1	1.8
Biotope-B	8	*Platycephalus indicus*	126.8	6.7	1.3	0.3
Biotope-B	9	*Argyrosomus argentatus*	104.2	21.7	1.0	0.9
Biotope-B	10	*Triaenopogon barbatus*	72.5	5.0	0.7	0.2
Biotope-B	11	*Thryssa mystax*	64.2	106.7	0.6	4.3
Biotope-B	12	*Odontamblyopus rubicundus*	4.7	1.7	0.0	0.1
Biotope-D	1	*Sardinella zunasi*	2019.1	467.5	67.0	70.6
Biotope-D	2	*Synechogobius hasta*	406.9	15.0	13.5	2.3
Biotope-D	3	*Amblychaeturichthys hexanema*	258.2	83.0	8.6	12.5
Biotope-D	4	*Konosirus punctatus*	178.0	25.5	5.9	3.9
Biotope-D	5	*Triaenopogon barbatus*	51.2	2.5	1.7	0.4
Biotope-D	6	*Cynoglossus semilaevis*	47.9	5.5	1.6	0.8
Biotope-D	7	*Thryssa kammalensis*	40.3	60.5	1.3	9.1
Biotope-D	8	*Platycephalus indicus*	9.8	1.5	0.3	0.2
Biotope-D	9	*Enedrias fangi*	2.2	0.5	0.1	0.1
Biotope-D	10	*Argyrosomus argentatus*	1.0	0.5	0.0	0.1
Biotope-H	1	*Synechogobius hasta*	3211.1	202.5	29.8	15.0

Biotope-H	2	*Amblychaeturichthys hexanema*	3108.9	714.2	28.9	52.8
Biotope-H	3	*Cynoglossus semilaevis*	1202.2	125.0	11.2	9.2
Biotope-H	4	*Platycephalus indicus*	1060.4	12.5	9.8	0.9
Biotope-H	5	*Cynoglossus joyneri*	695.9	10.8	6.5	0.8
Biotope-H	6	*Konosirus punctatus*	394.5	22.5	3.7	1.7
Biotope-H	7	*Setipinna tenuifilis*	270.0	90.0	2.5	6.7
Biotope-H	8	*Odontamblyopus rubicundus*	322.1	37.5	3.0	2.8
Biotope-H	9	*Thryssa kammalensis*	151.9	92.5	1.4	6.8
Biotope-H	10	*Argyrosomus argentatus*	88.9	15.8	0.8	1.2
Biotope-H	11	*Thryssa mystax*	69.9	18.3	0.6	1.4
Biotope-H	12	*Sillago japonica*	63.4	1.7	0.6	0.1
Biotope-H	13	*Pampus echinogaster*	47.7	1.7	0.4	0.1
Biotope-H	14	*Sardinella zunasi*	38.9	5.0	0.4	0.4
Biotope-H	15	*Eupleurogrammus muticus*	31.8	1.7	0.3	0.1
Biotope-H	16	*Triaenopogon barbatus*	10.8	0.8	0.1	0.1
Biotope-C	1	*Platycephalus indicus*	1475.2	23.3	22.8	2.8
Biotope-C	2	*Konosirus punctatus*	1369.7	103.3	21.2	12.6
Biotope-C	3	*Amblychaeturichthys hexanema*	977.7	236.7	15.1	28.7
Biotope-C	4	*Thryssa mystax*	744.4	113.3	11.5	13.8
Biotope-C	5	*Setipinna tenuifilis*	504.9	53.3	7.8	6.5
Biotope-C	6	*Thryssa kammalensis*	458.5	206.7	7.1	25.1
Biotope-C	7	*Synechogobius hasta*	375.9	13.3	5.8	1.6
Biotope-C	8	*Cynoglossus semilaevis*	359.1	26.7	5.6	3.2
Biotope-C	9	*Sardinella zunasi*	170.0	26.7	2.6	3.2
Biotope-C	10	*Odontamblyopus rubicundus*	10.7	13.3	0.2	1.6
Biotope-C	11	*Argyrosomus argentatus*	9.8	6.7	0.2	0.8

Biotope-S	1	*Konosirus punctatus*	5277.8	410.0	49.4	21.6
Biotope-S	2	*Amblychaeturichthys hexanema*	1653.1	310.0	15.5	16.3
Biotope-S	3	*Sardinella zunasi*	1382.2	450.0	12.9	23.7
Biotope-S	4	*Thryssa kammalensis*	902.1	630.0	8.4	33.2
Biotope-S	5	*Argyrosomus argentatus*	488.3	20.0	4.6	1.1
Biotope-S	6	*Platycephalus indicus*	339.8	10.0	3.2	0.5
Biotope-S	7	*Cynoglossus semilaevis*	248.8	25.0	2.3	1.3
Biotope-S	8	*Pampus echinogaster*	193.8	5.0	1.8	0.3
Biotope-S	9	*Setipinna tenuifilis*	179.1	30.0	1.7	1.6

Table S2. T-test results for fish $\delta^{15}N$

		Levene's Test for Equality of Variances		t-test for Equality of Means						
									95% CI of the Difference	
		F	Sig.	t	df	Sig. (2-tailed)	Mean Difference	Std. Deviation	Lower	Upper
B vs D	Equal variances assumed	0.697	0.407	1.898	61.000	0.062	0.39691	0.20911	-0.02123	0.81505
	Equal variances not assumed			1.883	57.225	0.065	0.39691	0.21075	-0.02507	0.81889
B vs H	Equal variances assumed	7.870	0.006	-0.355	76.000	0.724	-0.10754	0.30292	-0.71085	0.49578
	Equal variances not assumed			-0.392	66.449	0.696	-0.10754	0.27436	-0.65523	0.44016
B vs C	Equal variances assumed	3.396	0.070	-2.383	64.000	0.020	-0.59485	0.24959	-1.09346	-0.09624
	Equal variances not assumed			-2.383	53.823	0.021	-0.59485	0.24959	-1.09528	-0.09442
B vs S	Equal variances assumed	7.935	0.007	-9.795	58.000	0.000	-2.82502	0.28841	-3.40232	-2.24771
	Equal variances not assumed			-9.255	37.854	0.000	-2.82502	0.30525	-3.44304	-2.20700
D vs H	Equal variances assumed	4.707	0.033	-1.559	73.000	0.123	-0.50444	0.32349	-1.14917	0.14028
	Equal variances not assumed			-1.736	71.105	0.087	-0.50444	0.29065	-1.08398	0.07509
D vs C	Equal variances assumed	1.241	0.270	-3.656	61.000	0.001	-0.99176	0.27124	-1.53413	-0.44939
	Equal variances not assumed			-3.709	58.629	0.000	-0.99176	0.26740	-1.52690	-0.45662

D vs S	Equal variances assumed	4.259	0.044	-10.307	55.000	0.000	-3.22193	0.31261	-3.84841	-2.59544
	Equal variances not assumed			-10.069	42.882	0.000	-3.22193	0.31998	-3.86727	-2.57658
H vs C	Equal variances assumed	1.500	0.224	-1.459	76.000	0.149	-0.48731	0.33396	-1.15246	0.17783
	Equal variances not assumed			-1.523	75.925	0.132	-0.48731	0.31994	-1.12453	0.14990
H vs S	Equal variances assumed	0.090	0.765	-7.222	70.000	0.000	-2.71748	0.37626	-3.46791	-1.96705
	Equal variances not assumed			-7.445	60.130	0.000	-2.71748	0.36502	-3.44760	-1.98736
C vs S	Equal variances assumed	0.853	0.359	-6.537	58.000	0.000	-2.23017	0.34116	-2.91307	-1.54726
	Equal variances not assumed			-6.431	51.274	0.000	-2.23017	0.34679	-2.92629	-1.53405

Table S3. T-test results for fish TL

		Levene's Test for Equality of Variances		t-test for Equality of Means						
									95% CI of the Difference	
		F	Sig.	t	df	Sig. (2-tailed)	Mean Difference	Std. Deviation	Lower	Upper
B vs D	Equal variances assumed	0.733	0.395	1.912	61.000	0.061	0.11761	0.06151	-0.00539	0.24060
	Equal variances not assumed			1.897	57.229	0.063	0.11761	0.06199	-0.00652	0.24173
B vs H	Equal variances assumed	7.944	0.006	-0.357	76.000	0.722	-0.03184	0.08919	-0.20947	0.14579
	Equal variances not assumed			-0.394	66.417	0.695	-0.03184	0.08077	-0.19308	0.12940
B vs C	Equal variances assumed	3.380	0.071	-2.383	64.000	0.020	-0.17485	0.07338	-0.32145	-0.02825
	Equal variances not assumed			-2.383	53.846	0.021	-0.17485	0.07338	-0.32198	-0.02771
B vs S	Equal variances assumed	8.000	0.006	-9.777	58.000	0.000	-0.82976	0.08487	-0.99965	-0.65988
	Equal variances not assumed			-9.237	37.845	0.000	-0.82976	0.08983	-1.01163	-0.64789
D vs H	Equal variances assumed	4.725	0.033	-1.569	73.000	0.121	-0.14944	0.09524	-0.33926	0.04037
	Equal variances not assumed			-1.747	71.083	0.085	-0.14944	0.08556	-0.32004	0.02115
D vs C	Equal variances assumed	1.209	0.276	-3.667	61.000	0.001	-0.29245	0.07975	-0.45192	-0.13299
	Equal variances not assumed			-3.720	58.641	0.000	-0.29245	0.07862	-0.44980	-0.13511

D vs S	Equal variances assumed	4.269	0.044	-10.299	55.000	0.000	-0.94737	0.09198	-1.13171	-0.76303
	Equal variances not assumed			-10.062	42.867	0.000	-0.94737	0.09416	-1.17270	-0.75747
H vs C	Equal variances assumed	1.537	0.219	-1.455	76.000	0.150	-0.14301	0.09829	-0.33877	0.05275
	Equal variances not assumed			-1.519	75.933	0.133	-0.14301	0.09414	-0.33051	0.04449
H vs S	Equal variances assumed	0.092	0.762	-7.203	70.000	0.000	-0.79793	0.11078	-1.01886	-0.57699
	Equal variances not assumed			-7.426	60.154	0.000	-0.79793	0.10745	-1.01285	-0.58300
C vs S	Equal variances assumed	0.875	0.353	-6.526	58.000	0.000	-0.65492	0.10035	-0.85578	-0.45405
	Equal variances not assumed			-6.420	51.239	0.000	-0.65492	0.10202	-0.85970	-0.45013

Table S4. Proportion confidence intervals of food source contribution

	Biotope-H	Proportion confidence interval					
No.	Sample name	95% CI		75% CI		50% CI	
1	POM	0.00	0.37	0.01	0.26	0.02	0.18
2	SOM	0.00	0.24	0.00	0.14	0.01	0.09
3	Macroalgae	0.01	0.44	0.09	0.36	0.16	0.31
4	Cordgrass	0.04	0.43	0.12	0.36	0.17	0.31
5	YROM	0.10	0.40	0.17	0.35	0.21	0.31

	Biotope-C	Proportion confidence interval					
No.	Sample name	95% CI		75% CI		50% CI	
1	POM	0.00	0.37	0.01	0.26	0.01	0.17
2	SOM	0.00	0.12	0.00	0.06	0.00	0.03
3	Macroalgae	0.00	0.47	0.07	0.37	0.15	0.32
4	Cordgrass	0.03	0.44	0.13	0.38	0.18	0.32
5	YROM	0.17	0.43	0.23	0.39	0.26	0.36

	Biotope-B	Proportion confidence interval					
No.	Sample name	95% CI		75% CI		50% CI	
1	POM	0.04	0.38	0.15	0.34	0.21	0.31
2	SOM	0.16	0.19	0.19	0.30	0.21	0.27
3	Macroalgae	0.32	0.54	0.13	0.42	0.19	0.35
4	Cordgrass	0.04	0.42	0.12	0.35	0.18	0.31

	Biotope-D	Proportion confidence interval					
No.	Sample name	95% CI		75% CI		50% CI	
1	POM	0.26	0.79	0.40	0.70	0.48	0.65
2	SOM	0.17	0.53	0.25	0.45	0.30	0.41
3	Macroalgae	0.00	0.18	0.00	0.10	0.00	0.06
4	Cordgrass	0.00	0.11	0.00	0.06	0.00	0.04

	Biotope-S	Proportion confidence interval					
No.	Sample name	95% CI		75% CI		50% CI	
1	POM	0.00	0.61	0.11	0.48	0.21	0.41
2	SOM	0.16	0.54	0.25	0.46	0.29	0.41
3	Macroalgae	0.00	0.41	0.53	0.34	0.14	0.32
4	Cordgrass	0.00	0.28	0.00	0.18	0.01	0.12

Table S5. Mean contributions for fishes

	Biotope-H	Mean contributions				
No.	Species	POM	SOM	Macroalgae	Cordgrass	YROM
1	*Argyrosomus argentatus*	0.11	0.05	0.33	0.30	0.21
2	*Konosirus punctatus*	0.24	0.17	0.21	0.19	0.19
3	*Cynoglossus semilaevis*	0.17	0.10	0.29	0.30	0.13
4	*Thryssa kammalensis*	0.17	0.28	0.10	0.08	0.37
5	*Amblychaeturichthys hexanema*	0.18	0.11	0.28	0.25	0.18
6	*Sardinella zunasi*	0.25	0.22	0.18	0.15	0.19
7	*Platycephalus indicus*	0.23	0.17	0.24	0.22	0.14

	Biotope-C	Mean contributions				
No.	Species	POM	SOM	Macroalgae	Cordgrass	YROM
1	*Argyrosomus argentatus*	0.17	0.07	0.32	0.33	0.12
2	*Konosirus punctatus*	0.23	0.15	0.24	0.23	0.15
3	*Cynoglossus semilaevis*	0.22	0.08	0.26	0.22	0.21
4	*Thryssa kammalensis*	0.25	0.15	0.17	0.15	0.29
5	*Amblychaeturichthys hexanema*	0.22	0.08	0.27	0.24	0.20
6	*Sardinella zunasi*	0.18	0.05	0.29	0.25	0.23
7	*Platycephalus indicus*	0.24	0.16	0.23	0.21	0.17

	Biotope-B	Mean contributions			
No.	Species	POM	SOM	Macroalgae	Cordgrass
1	*Argyrosomus argentatus*	0.03	0.43	0.06	0.48
2	*Konosirus punctatus*	0.30	0.15	0.30	0.24
3	*Cynoglossus semilaevis*	0.32	0.15	0.30	0.23
4	*Thryssa kammalensis*	0.28	0.16	0.30	0.26
5	*Amblychaeturichthys hexanema*	0.31	0.19	0.27	0.22
6	*Sardinella zunasi*	0.29	0.33	0.20	0.18
7	*Platycephalus indicus*	0.27	0.21	0.27	0.25

	Biotope-D	Mean contributions			
No.	Species	POM	SOM	Macroalgae	Cordgrass
1	*Argyrosomus argentatus*	0.48	0.20	0.20	0.13
2	*Konosirus punctatus*	0.76	0.17	0.05	0.02
3	*Cynoglossus semilaevis*	0.67	0.13	0.13	0.07
4	*Thryssa kammalensis*	0.59	0.14	0.17	0.10
5	*Amblychaeturichthys hexanema*	0.58	0.18	0.15	0.09
6	*Sardinella zunasi*	0.25	0.54	0.12	0.09
7	*Platycephalus indicus*	0.20	0.62	0.10	0.08

	Biotope-S	Mean contributions			
No.	Species	POM	SOM	Macroalgae	Cordgrass

1	*Argyrosomus argentatus*	0.41	0.32	0.16	0.11
2	*Konosirus punctatus*	0.41	0.21	0.23	0.15
3	*Cynoglossus semilaevis*	0.32	0.27	0.23	0.18
4	*Thryssa kammalensis*	0.57	0.14	0.21	0.01
5	*Amblychaeturichthys hexanema*	0.31	0.24	0.24	0.20
6	*Sardinella zunasi*	0.33	0.41	0.15	0.11
7	*Platycephalus indicus*	0.32	0.20	0.27	0.22

Table S6. TL gaps between Biotope-S and other biotopes

	TL	TL_b	TL_n	SD(TL)	SD(TL_b)	SD(TL_n)
Biotope-B	3.30	3.33	3.15	0.22	0.22	0.24
Biotope-D	3.18	3.43	3.03	0.26	0.26	0.27
Biotope-H	3.33	3.43	3.21	0.47	0.47	0.47
Biotope-C	3.47	3.37	3.35	0.36	0.36	0.41
Biotope-S	4.13	3.76	4.23	0.42	0.42	0.61
S - B	0.83	0.42	1.08			
S - D	0.95	0.32	1.20			
S - H	0.80	0.33	1.02			
S - C	0.65	0.39	0.88			

Figure S1. $\delta^{13}C$ and $\delta^{15}N$ dot plots of fishes in five biotopes (C, D, H, C, S) and potential food sources (YROM = organic matter from the Yellow River, SOM = sedimental organic matter, POM = suspended particulate organic matter).

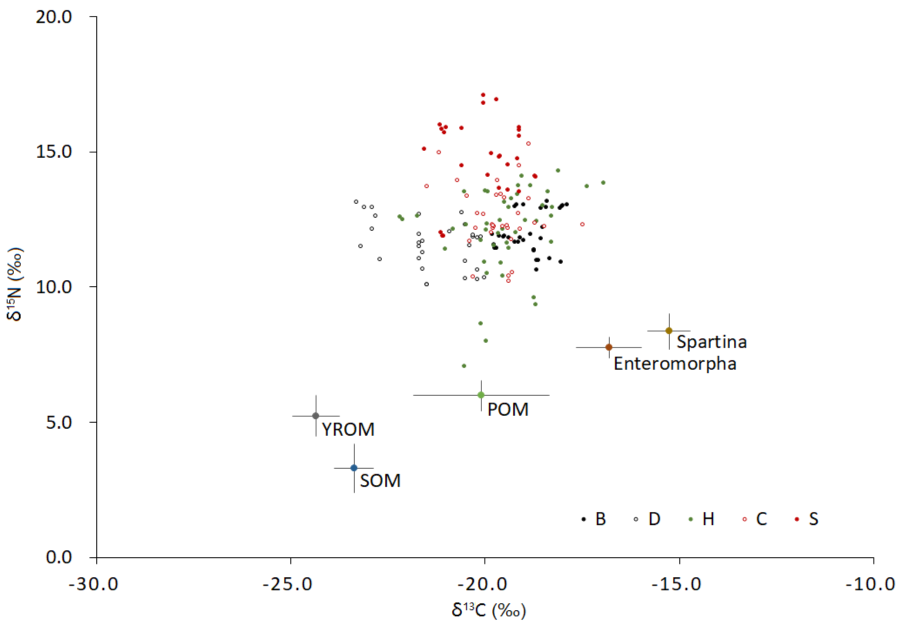

Supplement: Supplementary file 1 — Appendix S1 [file ECE3-11-11077-s001.pdf]
